# Inflammatory indexes as predictive factors for platinum sensitivity and as prognostic factors in recurrent epithelial ovarian cancer patients: a MITO24 retrospective study

**DOI:** 10.1038/s41598-020-75316-x

**Published:** 2020-10-23

**Authors:** Alberto Farolfi, Emanuela Scarpi, Filippo Greco, Alice Bergamini, Lucia Longo, Sandro Pignata, Claudia Casanova, Gennaro Cormio, Alessandra Bologna, Michele Orditura, Laura Zavallone, Laura Attademo, Valentina Gallà, Elisena Franzese, Eva Pigozzi, Vera Loizzi, Giorgio Giorda, Donatella Giardina, Raffaella Cioffi, Ugo De Giorgi

**Affiliations:** 1 Medical Oncology Department, Istituto Scientifico Romagnolo per lo Studio e la Cura dei Tumori (IRST) IRCCS, Meldola, Italy; 2 Unit of Biostatistics and Clinical Trials, Istituto Scientifico Romagnolo per lo Studio e la Cura dei Tumori (IRST) IRCCS, Meldola, Italy; 3 Medical Oncology Unit, ULSS, 9 Regione Veneto, Legnago, Italy; 4 Department of Obstetrics and Gynaecology, San Raffaele Scientific Institute, Milan, Italy; 5 Medical Oncology Unit, Ramazzini Hospital, Carpi, Italy; 6 Department of Urology and Gynecology, Istituto Nazionale Tumori IRCCS Fondazione G. Pascale, Napoli, Italy; 7 Department of Medical Oncology, Santa Maria Delle Croci Hospital, Ravenna, Italy; 8 Gynecology Oncology Unit, Università degli Studi di Bari & IRCCS Istituto Oncologico “Giovanni Paolo II”, Bari, Italy; 9 Medical Oncology Unit, Clinical Cancer Centre, IRCCS-Arcispedale S. Maria Nuova, Reggio Emilia, Italy; 10 Department of Clinical and Experimental Medicine “F. Magrassi”, Università Degli Studi Della Campania “Luigi Vanvitelli”, Naples, Italy; 11 Department Medical Oncology, Infermi Hospital, Biella, Italy; 12 Department of Gynecological Oncology, Centro Di Riferimento Oncologico (CRO) IRCCS, Aviano, Italy

**Keywords:** Ovarian cancer, Ovarian cancer

## Abstract

Neutrophil-to-lymphocyte ratio (NLR) and systemic inflammatory index (SII) are prognostic factors in epithelial ovarian cancer (EOC). Their predictive value for platinum-sensitivity and their role in recurrent EOC are unknown. A total of 375 EOC patients were retrospectively analyzed. The correlation between baseline NLR and SII, and platinum-free interval (PFI) according to first line bevacizumab treatment were analyzed using logistic regression analyses adjusted for baseline patient characteristics. Subsequently NLR and SII calculated before second line treatment initiation were evaluated to identify a potential correlation with progression-free survival (PFS) and overall survival (OS) in platinum-sensitive and in platinum-resistant population. In multivariate analysis, NLR ≥ 3 is an independent predictive factor for PFI at 6 months in the chemotherapy group (OR = 2.77, 95% CI 1.38–5.56, *p* = 0.004), not in bevacizumab treated patients. After having adjusted for ECOG performance status, histology, ascites, bevacizumab treatment at second line and BRCA status, NLR ≥ 3 and SII ≥ 730 are significantly associated with worse OS in platinum-sensitive (HR = 2.69, 95% CI 1.60–4.53, *p* = 0.002; HR = 2.11, 95% CI 1.29–3.43, *p* = 0.003, respectively), not in platinum-resistant EOC patients. Low NLR is an independent predictive factor for platinum-sensitivity in patients treated without bevacizumab. NLR and SII are prognostic factors in recurrent platinum-sensitive EOC patients.

## Introduction

Epithelial ovarian cancer (EOC) is the second most lethal tumour from gynaecological malignancies^1^. EOC is not a single entity and different histological subtypes, including low-grade serous, endometrioid, clear-cell, and mucinous ovarian cancers, may arise from ovarian epithelium^2^. Among these, high-grade serous ovarian cancer (HGSOC) is the most common histology of ovarian neoplasm, accounting for about 70% of cases and causing the majority (90%) of ovarian cancer deaths.

Several prognostic factors have been proposed to reliably predict EOC outcome, including histology, tumour stage, residual disease after surgical debulking, response to chemotherapy and BRCA1/2-mutation status^3^. Indeed, HGSOC is frequently associated with homologous repair (HR) deficiencies whereas microsatellite instability (MSI) phenotype has been reported in up to 14% of ovarian endometrioid carcinomas and in about 10% of clear cell ovarian cancer.

A gene expression analysis of endometrioid ovarian cancer and HGSOC identified four distinct molecular subtypes (“immunoreactive,” “differentiated,” “proliferative,” and “mesenchymal”) that did not have, however, a survival time significantly different^4^. A reanalysis of the TCGA classification on a Mayo Clinic cohort of HGSOC indicated the longest survival for the immunoreactive subtype^5^. However, this classification needs to be validated and the authors were not able to define the predictive role of each subtype^5^. In this context, it was previously reported that a high presence of tumour infiltrating lymphocytes (TILs), especially intraepithelial CD4+ and CD8+, correlates significantly with improved outcome^6–8^.

As opposed to patients that exhibit a robust immune response in terms of TILs presence and display a better prognosis, tumour immune-escape (a mechanism by which antitumor immunity is effectively neutralized) is regarded as one of the main reasons for disease progression and treatment failure. Tumour cells, immune-suppressive T regulatory cells (FOXP3+ CD4+), tumour-associated macrophages (TAMs) are responsible of the inhibition of the activity of immune effectors cells within the tumour microenvironment, including CD4+ T cells, CD8+ T cells, and NK cells, through the concerted action of a plethora of mediators comprising cytokines (such as IL-10, TGF-β, PGE2) and membrane-bound ligands including B7-H1 and programmed cells death protein 1 (PD-1)^9^. In this context, neutrophils can exert effects that might either be tumour promoting or tumour suppressive, depending on the context. Early-stage EOCs secrete factors that stimulate influx of neutrophils into the premetastatic omental niche and induce these neutrophils to form neutrophil extracellular traps (NETs). Cancer cells that are shed by tumours into the circulating peritoneal fluid become trapped by NETs and then form implants on the omentum^10^.

The neutrophil-to-lymphocyte ratio (NLR) (defined as the ratio of neutrophil to lymphocyte count) is the most widely used inflammatory marker to evaluate the systemic potential balance between neutrophil-dependent pro-tumour inflammation and lymphocyte-associated anti-tumour immune response^11–14^. Elevated NLR in EOC patients has been found to be associated with poor prognosis^15,16^. Again, more recent studies confirmed that a high NLR is correlated with an immunosuppressive profile^17^ and an NLR > 3 is associated to poorer overall survival and could be a predictive marker for treatment efficacy^8,18^.

Regardless all the prognostic factors evaluated till today, platinum-sensitivity, defined as patients who experience recurrence after 6 months from the end of primary platinum-based chemotherapy, is considered the main issue in treatment decision and a factor for predicting survival outcomes^19^. Since inflammatory indexes suggested to be associated with treatment efficacy^18^, we conducted further analyses within our multicenter retrospective study (Multicenter Italian Trials in Ovarian cancer and gynaecologic malignancies—MITO24) in order to evaluate if NLR and/or systemic inflammatory index (SII) are predictive for platinum-sensitivity and to evaluate if their prognostic value in recurrent EOC is confirmed regardless platinum-sensitivity.

## Results

### Inflammatory indexes as predictors of platinum sensitivity

Out of 375, 154 (41%) patients underwent primary debulking surgery without residual disease, 43 (11.5%) optimal cytoreduction (residual tumour < 10 mm), 88 (23.5% suboptimal debulking, and 90 (24%) neoadjuvant chemotherapy followed by interval surgery. At baseline, before first line treatment initiation, 220 (58.7%) has low NLR and 155 (41.3%) high NLR values, 156 (41.6%) and 219 (58.4%) has low and high SII values, respectively. 74 (19.7%) patients received as first line treatment chemotherapy plus bevacizumab (bevacizumab group) and 301 (80.3%) chemotherapy alone. Patients, divided into groups on the basis of marker cut-offs, are all comparable for histology, stage, BReast CAncer susceptibility gene (BRCA) status, and treatment. Patients with NLR ≥ 3 have more frequently an ECOG performance status of 1–2 and ascites. Even the high SII group has a higher ECOG performance status and are older at diagnosis. Table 1 represents baseline patient characteristics by inflammatory index.

**Table 1:** Baseline patient characteristics by NLR and SII status.

Patient characteristics	NLR		*p*	SII		*p*
	< 3	≥ 3		< 730	≥ 730	
	No. (%)	No. (%)		No. (%)	No. (%)	
**Median age, years** [range]	60 [19–83]	64 [32–85]	0.066	58 [31–83]	64 [19–85]	0.012
**Performance Status** (ECOG)						
0	147 (68.7)	79 (51.3)	0.0007	111 (73.5)	115 (53.0)	< 0.0001
1–2	67 (31.3)	75 (48.7)		40 (26.5)	102 (47.0)	
**EOC histology**						
Other	65 (29.5)	49 (31.6)	0.669	51 (32.7)	63 (28.8)	0.416
Serous	155 (70.5)	106 (68.4)		105 (67.3)	156 (71.2)	
**Stage**						
III	163 (74.1)	102 (65.8)	0.083	118 (75.6)	147 (67.1)	0.074
IV	57 (25.9)	53 (34.2)		38 (24.4)	72 (32.9)	
**Ascites**						
No	113 (53.0)	66 (42.6)	0.047	83 (54.2)	96 (44.6)	0.070
Yes	100 (47.0)	89 (57.4)		70 (47.8)	119 (55.4)	
**BRCA status**						
Wild type	42 (63.6)	21 (80.8)	0.113	31 (63.3)	32 (74.4)	0.253
Mutated	24 (36.4)	5 (19.2)		18 (36.7)	11 (25.6)	
Unknown	154	129		107	176	
**Treatment**						
CT	181 (82.3)	120 (77.4)		124 (79.5)	177 (80.8)	
CTB	39 (17.7)	35 (22.6)	0.245	32 (20.5)	42 (19.2)	0.749

BRCA, BReast CAncer susceptibility gene; CT, chemotherapy; CTB, chemotherapy with bevacizumab; NLR, neutrophil-to-lymphocyte ratio; SII, systemic immune-inflammation index.

In multivariate analysis adjusted for CA125 serum marker (as continuous variable), ECOG performance status (0–1 vs. 2), histology (serous vs. others), ascites (absent vs. present), bevacizumab treatment, and inflammatory indexes, CA125 (HR = 1.29, 95% CI 1.16–1.42 for PFS and HR = 1.31, 95% CI 1.13–1.51 for OS), performance status (HR = 4.5, 95% CI 2.07–9.75 for PFS; HR = 3.15, 95% CI 1.17–8.46 for OS), NLR (HR = 1.23, 95% CI 1.10–1.37 for PFS; HR = 1.41, 95% CI 1.23–1.62 for OS) and SII (HR = 1.00, 95% CI 1.00–1.01 for PFS; HR = 1.00, 95% CI 1.00–1.01 for OS) demonstrate to be significantly associated with patients outcome.

In univariate analysis, patients with high NLR (≥ 3) and SII (≥ 730) has a significantly probability of shorter PFI at 6 and 12 months in overall cohort. In multivariate analyses adjusted for ECOG performance status, histology, stage and ascites, only NLR is an independent predictive factor for PFI at 6 months (OR = 2.52, 95% CI 1.30–4.87, *p* = 0.006) and at 12 months (OR = 2.05, 95% CI 1.05–4.01, *p* = 0.036) in the overall population and in the chemotherapy group (OR = 2.77, 95% CI 1.38–5.56, *p* = 0.004; HR = 2.27, 95% CI 1.10–4.70, *p* = 0.027, respectively). Inflammatory indexes are not predictive of platinum-sensitivity in the bevacizumab group (Table 2).

**Table 2:** Multivariate analyses of inflammatory indexes as predictors of platinum-free interval.

	PFI (< 6 months)		PFI (< 12 months)	
	OR (95% CI)*	p*	OR (95% CI)*	p*
**Overall population**				
**NLR**				
** < 3**	1.00	0.006	1.00	0.036
** ≥ 3**	2.52 (1.30–4.87)		2.05 (1.05–4.01)	
**SII**				
** < 730**	1.00	0.413	1.00	0.786
** ≥ 730**	0.74 (0.36–1.53)		0.91 (0.45–1.84)	
**CT group**				
**NLR**				
** < 3**	1.00	0.004	1.00	0.027
** ≥ 3**	2.77 (1.38–5.56)		2.27 (1.10–4.70)	
**SII**				
** < 730**	1.00	0.498	1.00	0.663
** ≥ 730**	0.76 (0.35–1.67)		0.84 (0.39–1.82)	
**CTB group**				
**NLR**				
** < 3**	1.00	0.538	1.00	0.774
** ≥ 3**	0.47 (0.04–5.15)		0.75 (0.11–5.25)	
**SII**				
** < 730**	1.00	0.696	1.00	0.599
** ≥ 730**	1.65 (0.13–20.56)		1.78 (0.21–15.14)	

CI, confidence interval; CT, chemotherapy; CTB, chemotherapy with bevacizumab; NLR, neutrophil-to-lymphocyte ratio; OR, odd ratio; PFI, platinum-free interval; SII, systemic immune-inflammation index.

*Adjusted for ECOG performance status, histology, stage and ascites.

### Relapsed ovarian cancer patients’ characteristics

Information on inflammatory index levels of relapsed EOC was available for 222 patients. Of these, 156 (70.3%) and 66 (29.7%) has low and high NLR values, 140 (63.1%) and 82 (36.9%) low and high SII values, respectively. Out 222 patients, 28 (12.6%) relapsed EOC patients were treated with chemotherapy plus bevacizumab and 194 (87.4%) with chemotherapy alone as second line therapy. Information about platinum-sensitivity was available in 216. Of these 156 (72.2%) patients were defined as platinum-sensitive and 60 (27.8%) were platinum-resistant. Patients’ characteristics by inflammatory index status (high versus low) are all comparable for the main clinical features, platinum-sensitivity and treatment, as shown in Table 3.

**Table 3:** Relapsed ovarian cancer patient characteristics at second line (*n* = *2*22*).*

Patient characteristics	NLR			SII		*p*
	< 3	≥ 3		< 730	≥ 730	
	No. (%)	No. (%)	*p*	No. (%)	No. (%)	
**Median age, years** [range]	60 (32–82)	61 (40–83)	0.519	60 (32–82)	60 (36–83)	0.744
**Performance Status** (ECOG)						
0	149 (95.5)	64 (97.0)	0.616	135 (96.4)	78 (95.1)	0.729
1–2	7 (4.5)	2 (3.0)		5 (3.6)	4 (4.9)	
**Ovarian cancer histology**						
High grade serous	110 (70.5)	48 (72.7)	0.740	103 (73.6)	55 (67.1)	0.303
Other	46 (29.5)	18 (27.3)		37 (26.4)	27 (32.9)	
**Ascites**						
No	74 (49.7)	33 (50.0)	0.964	66 (49.6)	41 (50.0)	0.957
Yes	75 (50.3)	33 (50.0)		67 (50.4)	41 (50.0)	
**BRCA status**						
Wild type	135 (86.5)	58 (87.9)	0.787	119 (85.0)	74 (90.2)	0.264
Mutated	21 (13.5)	8 (12.1)		21 (15.0)	8 (9.8)	
Unknown						
**Platinum sensitivity**						
No	40 (26.5)	20 (30.8)		34 (25.0)	26 (32.5)	
Yes	111 (73.5)	45 (69.2)	0.520	102 (75.0)	54 (67.5)	0.236
**Treatment at relapse**						
CT	135 (86.5)	59 (89.4)		118 (84.3)	76 (92.7)	
CTB	21 (13.5)	7 (10.6)	0.559	22 (15.7)	6 (7.3)	0.070

BRCA, BReast CAncer susceptibility gene; CT, chemotherapy; CTB, chemotherapy with bevacizumab; ECOG, Eastern Cooperative Oncology Group; NLR, neutrophil-to-lymphocyte ratio; SII, systemic immune-inflammation index.

Elevated NLR values are associated with a shorter median PFS (5.2 months, 95% CI 3.0–8.6 vs. 9.7 months, 95% CI 8.1–11.1, *p* = 0.001; HR 1.93, 95% CI 1.28–2.91) and OS (14.6 months, 95% CI 6.5–24.4 vs. 28.8 months, 95% CI 22.0–40.3, *p* = 0.0004; HR 2.37, 95% CI 1.44–3.8) in relapsed EOC patients treated without bevacizumab. Again, median PFS is significantly associated with SII levels in the chemotherapy-only group (5.8 months, 95% CI 4.3–8.3 vs.9.8 months, 95% CI 8.7–11.3, in high and low SII patients, respectively; *p* = 0.004; HR = 1.65, 95% CI 1.17–2.31), as median OS (17.2 months, 95% CI 10.3–23.8 vs. 33.3 months, 95% CI 23.7–42.8, in low and high SII patients, respectively; *p* < 0.0001; HR = 2.33, 95% CI 1.57–3.47). PFS and OS are not associated with NLR and SII in relapsed EOC patients treated with bevacizumab.

### Prognostic value of inflammatory indexes according to platinum sensitivity

Median follow-up of recurrent ovarian cancer patients treated with a second line therapy is 38 months (range 1–104), with a median PFS of 9.4 months (95% CI, 8.1–10.1) and a median OS of 24.7 months (95% CI, 21.4–32.8). As shown in Table 4, NLR is not associated with PFS in platinum-sensitive or in platinum-resistant group. SII is not related with PFS in platinum-resistant patients but is significantly associated with PFS in platinum-sensitive patients. In particular, median PFS is 12.6 (95% CI 10.2–14.0) and 10.1 (95%CI 8.1–11.2) months in patients with low and high SII, respectively (*p* = 0.046), with a HR of 1.46 (95% CI 1.00–2.12, *p* = 0.048). However, at multivariate analyses adjusted for ECOG performance status, histology, ascites, bevacizumab treatment at second line and BRCA status, HR is 1.44 (95% CI, 0.98–2.10; *p* = 0.061).

**Table 4:** Multivariate analysis of inflammatory indexes (NLR and SII) according to platinum sensitivity.

			PFS					OS			
	N. pts	N. events	Median PFS (months) (95% CI)	*p*	HR (95%CI)*	p*	N. events	Median OS (months) (95% CI)	*p*	HR (95% CI)*	p*
**Platinum-Resistant**											
**NLR < 3**	40	38	4.9 (3.0–6.2)	0.296	1.00	0.225	34	18.2 (13.1–23.8)	0.267	1.00	0.112
** ≥ 3**	20	20	3.0 (1.6–5.3)		1.46 (0.79–2.68)		13	14.6 (4.4–19.5)		1.83 (0.87–3.84)	
**SII < 730**	34	32	4.6 (2.8–6.2)	0.442	1.00	0.457	29	18.9 (14.6–23.8)	0.173	1.00	0.194
** ≥ 730**	26	26	3.4 (1.8–6.0)		1.25 (0.69–2.25)		18	14.6 (4.4–19.5)		1.59 (0.79–3.19)	
**Platinum-Sensitive**											
** NLR < 3**	111	95	11.5 (10.0–13.3)	0.439	1.00	0.260	49	42.8 (34.4–50.8)	0.0003	1.00	0.0002
** ≥ 3**	45	34	10.2 (8.3–13.6)		1.26 (0.84–1.90)		24	23.7 (16.5–32.8)		2.69 (1.60–4.53)	
** SII < 730**	102	87	12.6 (10.2–14.0)	0.046	1.00	0.061	45	42.8 (34.1–50.8)	0.002	1.00	0.003
** ≥ 730**	54	42	10.1 (8.1–11.2)		1.44 (0.98–2.10)		28	23.7 (13.8–40.4)		2.11 (1.29–3.43)	

CI, confidence interval; N, number of patients; NRL, neutrophil-to-lymphocyte ratio; PFS, progression free survival; OS, overall survival; SII, systemic immune-inflammation index.

*Adjusted for ECOG performance status, histology, ascites, bevacizumab treatment at second line and BRCA status.

**Figure 1:**
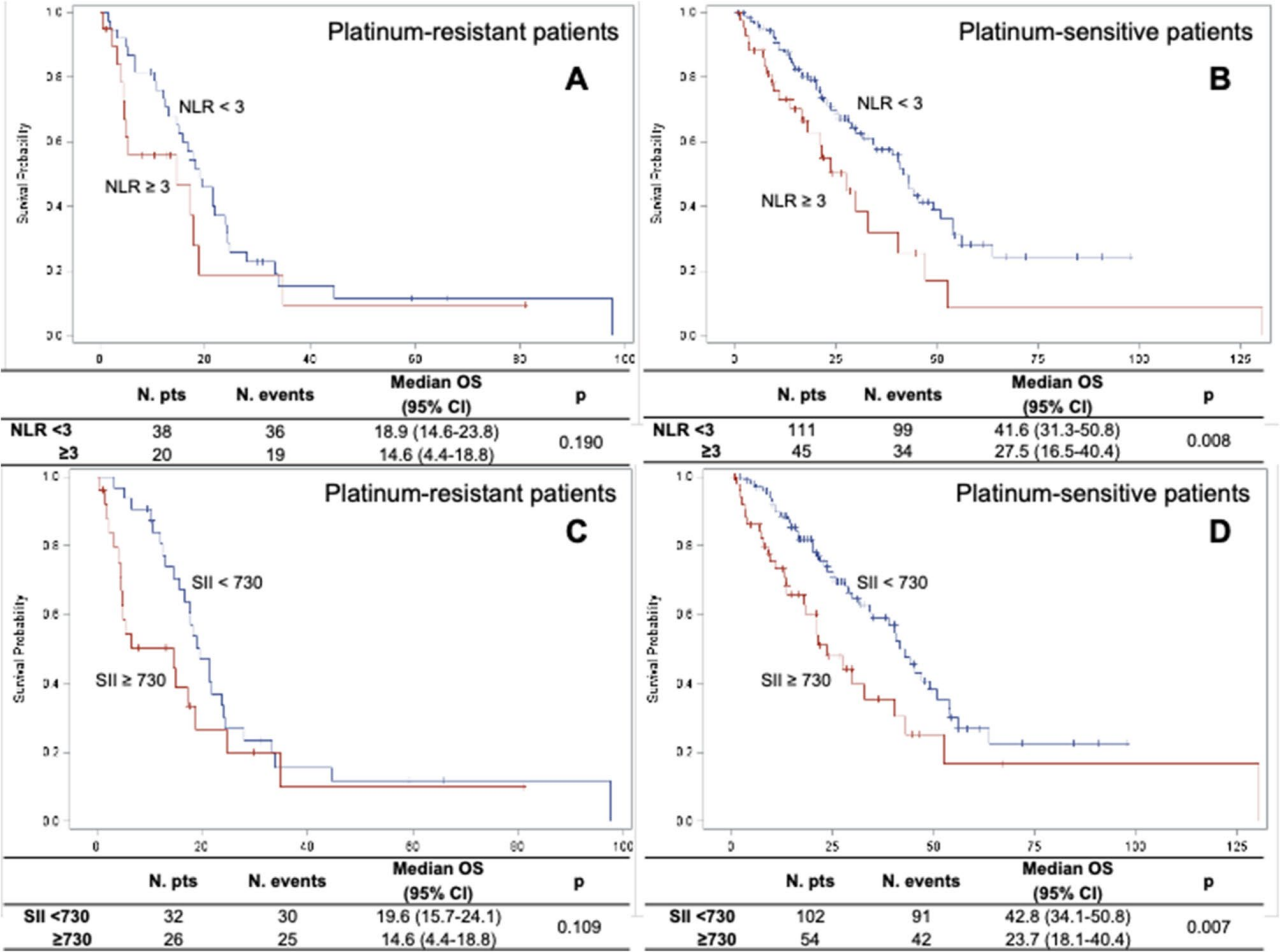
OS according to platinum sensitivity as a function of the neutrophil-to-lymphocyte ratio (NLR, **A**,**B**) and of the systemic inflammatory index (SII, C and D).

Again, NLR and SII are not associated with OS in platinum-resistant patients (Fig. 1A,B). Median OS is significantly associated with NLR levels in the platinum-sensitive group (42.8 vs. 23.7 months in low and high NLR patients, respectively, *p* = 0.0003) and with SII (42.8 vs. 23.7 months, in low and high SII patients, respectively, *p* = 0.0002) (Fig. 1C and 1D). In multivariate analysis adjusted for ECOG performance status, histology, ascites, bevacizumab treatment and BRCA status, both high NLR and high SII are significantly associated with worse OS in platinum-sensitive group (HR = 2.69, 95% CI 1.60–4.53, *p* = 0.0002; HR = 2.11, 95% CI 1.29–3.43, *p* = 0.003, respectively) (Table 4).

## Discussion

Tumour growth and progression is the results of a reciprocal interplay of cancer cells and host cells, where both the innate and adaptive immune cells create a tumour-promoting and immunosuppressive tumour microenvironment^9^. In this context, inflammation through the secretion of cytokines and chemokines facilitates angiogenesis and proliferation and prevents apoptosis, contributing in cancer progression^20^. Thus, tumour microenvironment exerts a fundamental role in tumour initiation, progression and metastatization. It was demonstrated, indeed, that early stage EOC actively recruits neutrophils creating a premetastatic niche in the omentum, where cancer cells might migrate facilitating the development of peritoneal carcinomatosis^10^.

Given the emerging role identified in tumour progression of innate and adaptive immune cells, inflammatory markers including NLR and SII have been studied in many solid tumours^11–14^ and have been found to be useful prognostic factors in EOC patients^21^. However, inflammatory indexes calculated using blood count may not directly reflect the tumour-promoting ability of neutrophils. Though, peritoneal carcinomatosis was strongly inhibited in neutrophil-depleted mice^10^, suggesting a potential role for inflammatory indexes to predict EOC outcome. In our study, we demonstrated, indeed, that NLR and SII are prognostic factors even in recurrent platinum-sensitive EOC, a biological condition more similar to the disease seen at first line.

Although most patients with EOC initially respond to platinum-based chemotherapy, the large majority of patients will relapse. About 20% of women experience disease progression ≤ 6 months after a platinum-based regimen completion (platinum-resistant) or relapse within 4 to 6 weeks after last cycle of first-line chemotherapy with a platinum agent (platinum-refractory)^22^. Many efforts have been made over the years to develop accurate predictive factors in EOC patients treated with platinum-based regimens. However, no validated predictive biomarkers have been found to help in determining likelihood of primary platinum-refractory or platinum-resistant disease that can be used in clinical practice^23^.

Since systemic inflammation induced by cancer cells anticipates tumour progression, inflammatory indexes seem to be useful to predict treatment efficacy as regard to therapy delivered^18^. Neutrophils produce tumour necrosis factor, interleukin 1 and interleukin 6, favouring cancer progression, and release VEGF, promoting adhesion and seeding of distant organ sites^24^. High platelets count, common in patients with EOC, is associated with patient prognosis^25^ and induces circulating tumour cell epithelial-mesenchymal transition, promoting extravasation to metastatic sites^26^. Lymphocytes exert a critical role in cancer-specific immune response by inducing cytotoxic cell death and inhibiting tumour cell proliferation and migration^6,27^.

In the present study we investigated the potential value of inflammatory indexes as predictive factors of platinum-sensitivity in patients with epithelial ovarian cancer. NLR < 3 was an independent predictive factor for platinum-sensitivity, when adjusted for ECOG performance status, histology, stage and ascites. Another recent study^28^, found that high NLR was an independent predictive factor for platinum-resistance, with a cut-off point value similar to the one used in our study. Interestingly, in our study in the sub-group of patients treated with bevacizumab as first line, NLR loses its predictive value. Similarly, we previously demonstrated that patients treated with bevacizumab as first line did not show a different PFS or OS in relation to the value of inflammatory indexes^18^ and we confirmed in our present study that NLR and SII are not associated with PFS and OS in relapsed EOC patients treated with bevacizumab.

However, PARP inhibitors are changing the treatment paradigm of ovarian cancer. BRCA, homologous recombination status and platinum-sensitivity are until now the only validated predictive markers^29–31^. Maybe, this is one of the major limitations of our study, because none of the patients received a first line maintenance treatment with a PARP inhibitor. However, as we previously demonstrated a significant correlation between inflammatory indexes and clinical outcome as a function of treatment, it would be very interesting if NLR and/or SII will be useful tools to predict PARP inhibitor maintenance therapy. Another limitation is the retrospective nature of our study, which may have led to bias in the data analysis. However, the results produced and the consistency with other similar research, invite to validate inflammatory indexes as prognostic and predictive factors.

In conclusion, low NLR is an independent predictive factor for platinum-sensitivity in epithelial ovarian cancer. Both high NLR and high SII are negative prognostic factors in recurrent platinum-sensitive ovarian cancer patients, independently by ECOG performance status, histology, ascites, bevacizumab treatment and BRCA status. Validation of these easy biomarkers is warranted.

## Patients and methods

### Patient population

Patients with advanced epithelial ovarian cancer (International Federation of Gynaecology and Obstetrics—FIGO stage III or IV) consecutively treated with first-line chemotherapy (with or without bevacizumab) from 1st January 2007 to 30th June 2015 were considered eligible for this multicenter, retrospective study. As previously reported^18^, patients were excluded if they had been treated with steroids or other immune-modulating agents within a month of starting chemotherapy, or had been diagnosed with infections or immune deficiencies.

Information on neutrophil, lymphocyte and platelet counts from blood tests carried out at baseline (immediately before the 1st cycle of the first line) and before 2nd line initiation was collected. SII was calculated as (platelet count × neutrophil count)/lymphocyte count, and NLR was obtained by dividing the absolute neutrophil count by the absolute lymphocyte count. NLR ≥ 3 and SII ≥ 730 were considered as high values, as previously determined^14,18^.

After treatment completion, patients were followed up with physical examination, radiographic evaluation (CT scan of the chest and abdomen) and CA125 blood test every 3–4 months in the first two years, and every 6 months thereafter. After the 5th years, patients were visited annually. Progression was defined as the appearance of a new lesion or the increase in dimension of a known metastasis according to the Response Evaluation Criteria in Solid Tumours. Increase in tumour marker alone was not considered a progressive disease (PD).

The study was approved, by the Ethics Committee of the coordinating centre (Comitato Etico Area Vasta Romagna e IRST) and by all participating centres (Comitato Etico Area Vasta Romagna e IRST, Comitato Etico Seconda Università degli Studi di Napoli—Azienda Ospedaliera Universitaria SUN—AORN "Ospedali dei Colli", Comitato Etico per le Sperimentazioni Cliniche delle Province di Verona e Rovigo, Comitato Etico Provinciale di Modena, Comitato Etico Provinciale di Reggio Emilia, Comitato Etico Interaziendale A.O.U. "Maggiore della Carità", ASL Bl, ASL NO, ASL VCO, Comitato Etico Indipendente IRCCS Pascale). The trial was carried out in accordance with good clinical practice guidelines and with the principles laid down in the 1964 Declaration of Helsinki. Patients who were still alive provided written informed consent. For dead patients, accordingly with Italian laws, all the best was done to obtain the consent from their Legal authorized representative or next of kin.

### Statistical analysis

The aim of the present study was to examine the predictive value of baseline inflammatory index levels in terms of platinum-free interval (PFI) as regard to bevacizumab treatment received. The second objective of these analyses was to evaluate the prognostic value of NLR and SII (calculated before second line initiation) in patients with recurrent ovarian cancer as regard to platinum-sensitivity.

PFI was defined as the time from the last dose of platinum to the first documented evidence of PD, per investigator assessment, or death from any cause. Patients who were not in progression at the time of the analysis were censored on the date of their last of tumour evaluation. Patients were defined as platinum sensitive if they had a recurrence after at least 6 months from the last dose of platinum.

Logistic regression analysis was used to estimate odd ratio (OR) and their 95% confidence interval (95% CI).

Progression-free survival (PFS) was defined as the time from the start of second line of chemotherapy to the first documented evidence of PD, per investigator assessment, or death from any cause. Patients who were not in progression at the time of the analysis were censored on the date of their last of tumour evaluation. Overall survival (OS) was defined as the time interval between start of chemotherapy and death from any cause. Patients who were no longer alive at the time of the analysis or had been lost to follow-up were censored on the date of their last follow-up visit. PFS and OS were estimated by the Kaplan–Meier method and curves were compared by the log rank test (at a significance level of 5%). Estimated hazard ratios (HRs) and their two-sided 95% confidence intervals (95% CI) were calculated using the Cox proportional-hazard model.

NLR ≥ 3 and SII ≥ 730 were considered as high values, as previously determined^10,14^. The chi-square test was used to evaluate the association between NLR and SII levels and baseline patient characteristics. All p values were based on two-sided testing and statistical analyses were performed using SAS statistical software version 9.4 (SAS Inc., Cary, NC, USA).
